# Application of mesenchymal stem cells for anti-senescence and clinical challenges

**DOI:** 10.1186/s13287-023-03497-z

**Published:** 2023-09-19

**Authors:** Yaping Wang, Tianyun Gao, Bin Wang

**Affiliations:** 1 Clinical Stem Cell Center, Nanjing Drum Tower Hospital Clinical College of Nanjing University of Chinese Medicine, Nanjing, 210008 People’s Republic of China; 2 Clinical Stem Cell Center, The Affiliated Drum Tower Hospital of Nanjing University Medical School, Nanjing, 210008 People’s Republic of China

**Keywords:** Senescence, Aging, Stem cells, Regenerative medicine, Mesenchymal stromal cells, Senescence-related diseases

## Abstract

Senescence is a hot topic nowadays, which shows the accumulation of senescent cells and inflammatory factors, leading to the occurrence of various senescence-related diseases. Although some methods have been identified to partly delay senescence, such as strengthening exercise, restricting diet, and some drugs, these only slow down the process of senescence and cannot fundamentally delay or even reverse senescence. Stem cell-based therapy is expected to be a potential effective way to alleviate or cure senescence-related disorders in the coming future. Mesenchymal stromal cells (MSCs) are the most widely used cell type in treating various diseases due to their potentials of self-replication and multidirectional differentiation, paracrine action, and immunoregulatory effects. Some biological characteristics of MSCs can be well targeted at the pathological features of aging. Therefore, MSC-based therapy is also a promising strategy to combat senescence-related diseases. Here we review the recent progresses of MSC-based therapies in the research of age-related diseases and the challenges in clinical application, proving further insight and reference for broad application prospects of MSCs in effectively combating senesce in the future.

## Introduction

Senescence is one of the hot topics at the moment, which is accompanied by the problem of aging population [1, 2]. Senescence has a variety of adverse effects on the body and is the most critical pathogenic factor of senile diseases. For example, senescence causes skin senescence. As a barrier of the body, skin can undergo aging or pathological changes with senescence [3], which may result from the accumulation of senescent cells. In addition, senescence can lead to the aging of the immune system, disrupting the homeostasis of macrophages [4] and increasing morbidity and mortality in the elderly [5]. Diabetes mellitus, especially type 2 mellitus, is a common disease associated with senescence [6]. With the progress of senescence, the incidence of some neurodegenerative diseases also increases, such as Alzheimer’s disease (AD) and Parkinson’s disease (PD). AD is a severe neurodegenerative disorder that is the most common form of dementia [7] as well as a senescence-related disease that increases exponentially with age [8]. PD is a common neurodegenerative disease characterized by degenerative death of dopaminergic neurons in the substantia nigra of the midbrain. And it is more common in the elderly, which has been proved to be a major senescence-related disease [9]. In addition, multiple sclerosis (MS) [10], obesity [11], cardiovascular diseases (CVDs) [12], and hematopoietic dysfunction [13] are also diseases associated with senescence. To date, researchers have been searching for effective treatments for senescence-associated diseases, such as appropriate nutritional intervention [14, 15], caloric restriction (CR) [16], dietary restriction (DR) [17], maintenance of iron homeostasis [18, 19], and drug therapy [20, 21]. However, the effect of these methods on delaying senescence or treating senescence-associated disorders is not very ideal. Therefore, we urgently need to seek a better way to delay or even reverse senescence. It is known that senescence is often accompanied by cellular senescence [22]. Accordingly, a connection between stem cells and senescence has been recently proposed, and cell-based therapy is emerging as an innovative approach for senescence, especially mesenchymal stromal cell (MSC)-based therapy. According to previous studies, stem cells have great advantages in the treatment of a variety of diseases, and they also have a certain effect on senescence. Currently, MSCs are promising candidates for stem cells therapy for senescence due to their unlimited proliferation and multiple differentiation potentials, migration and homing ability, and immunoregulation properties. In this review, we mainly summarize the role and latest progress of MSCs in the treatment of senescence, as well as the current status and challenges of MSCs in clinical research are also discussed, providing new insight into MSC-based therapy for anti-senescence and associated diseases.

## Overview of senescence

**Fig. 1:**
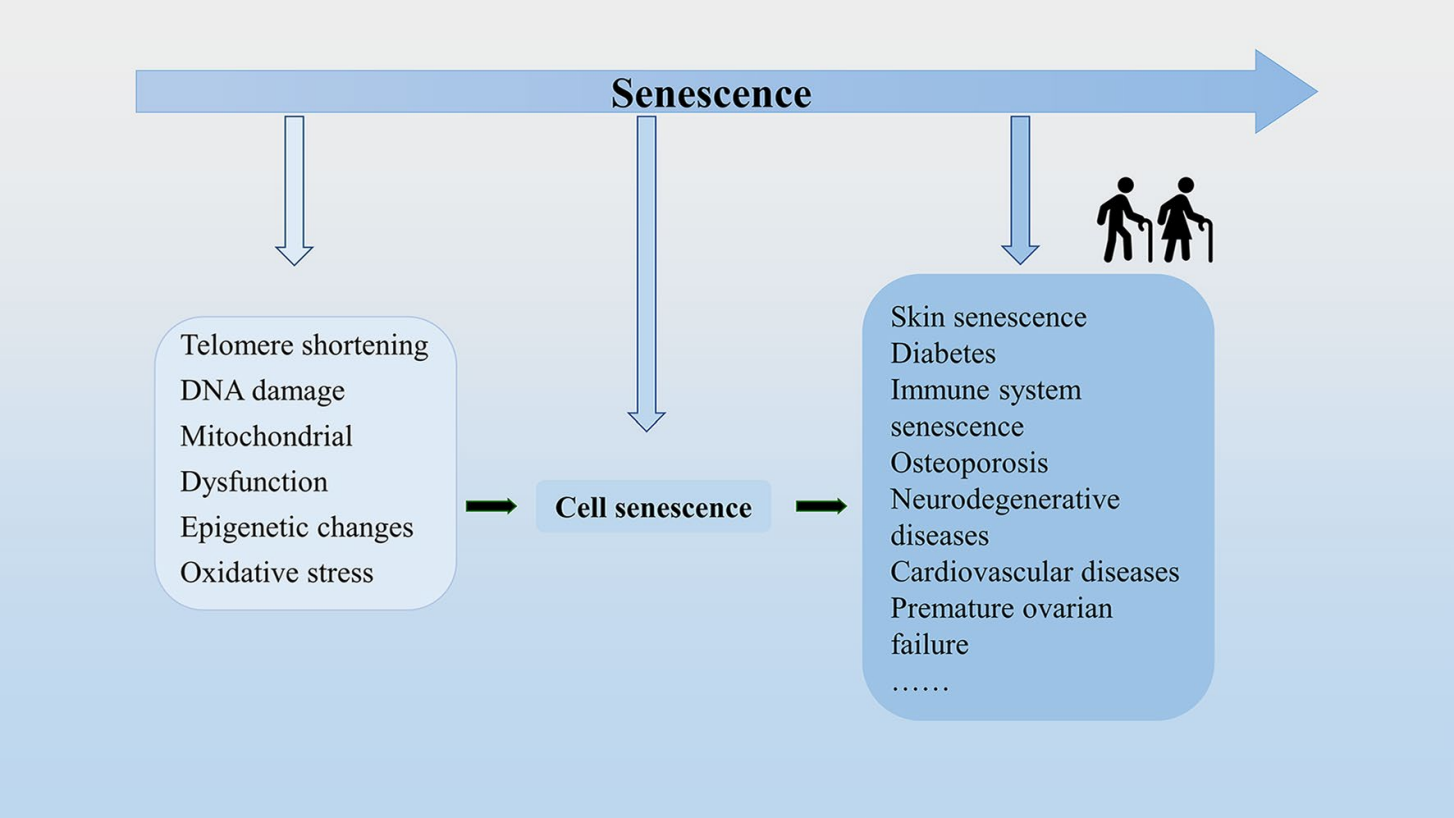
Senescence is often accompanied by cellular senescence, which caused by multiple factors, such as telomere shortening, DNA damage, mitochondrial dysfunction, epigenetic changes, and oxidative stress. And senescence leads to the occurrence of age-related diseases

Senescence occurs with age, which may be caused by multiple factors, such as telomere shortening [23, 24], DNA damage [25, 26], mitochondrial dysfunction [27], epigenetic changes [28–32], and oxidative stress [33]. All of these are interrelated, eventually leading to senescence. It has been proven that senescence is often accompanied by cellular senescence, which is a key mechanism of senescence [22] (Fig. 1). Evidence is mounting that intracellular reactive oxygen species (ROS) can trigger cellular senescence [34] Cellular senescence is a cell state triggered by stressful insults and certain physiological processes, which is characterized by cell cycle arrest [35], macromolecular modifications, the secretory phenotype and deregulated metabolism [36, 37]. Cell cycle arrest is a common feature of senescent cells and is often irreversibly arrested in the G1 or G2 phase [37, 38]. Macromolecular modifications include altered DNA methylation, aberrant histone modifications, loss of heterochromatin, disordered 3D genome architecture, and deregulated RNA modifications [38]. Senescence-associated secretory phenotypes (SASPs) refer to a large number of soluble factors synthesized and secreted by senescent cells, including pro-inflammatory cytokines, chemokines, angiogenic factors, growth modulators, and matrix metalloproteinases [37, 39–42]. As the number of senescent cells increases during senescence [43], it can promote some inflammations through SASP. SASP is a prominent feature of all senescent cells, which can trigger chronic inflammation to cause pro-aging consequences [36, 38], such as atherosclerosis, neurodegeneration, and myocardial fibrosis [44, 45]. This type of diseases has certain differences and connections with other senescence-related diseases. Other senescence-related diseases can also be caused by inflammation, but in addition to inflammation, there are other factors included. For example, in addition to inflammatory factors, decreased bone mineral density and increased bone fragility are also major factors for osteoporosis [46]. In addition, there are some senescence-related diseases caused by mutations or drugs. Deregulated metabolism is closely related to senescence, and researchers have verified seven age-related metabolites, such as NAD, AKG, tryptophan, methionine, spermidine, triglyceride, and cholesterol [38]. Deregulated metabolism can cause metabolic stress, which can be transmitted through metabolic signaling, through a variety of signaling pathways, and ultimately lead to aging [28]. Cellular senescence can be caused by a variety of factors. The types of cellular senescence include oncogene-induced senescence, replicative senescence, oxidative stress-induced senescence, therapy-induced senescence [36]. Oncogene-induced senescence can be caused by a variety of oncogenes, and it is associated with telomere dysfunction [47]. Replicative senescence is defined as the depletion of proliferative potential and irreversible growth arrest. It is mainly associated with telomere shortening during DNA replication [36]. When telomeres shorten to the Hayflick limit, irreversible cell cycle arrest is triggered. Oxidative stress-induced senescence manifests as cellular senescence caused by oxidative products or oxidants of cell metabolism. Senescence-related oxidative damage continues to accumulate in the body, exceeding the removal ability of body. These accumulated reactive oxygen species aggravate senescence-related DNA damage and accelerate cell senescence [48–50]. Therapy-induced senescence refers to the fact that anticancer drugs can induce the senescence of cancer cells [36, 51–53]. In conclusion, senescent cells have a great impact on the body and accelerate the process of aging. On the one hand, senescent cells will have reduced differentiation capacity. For example, the ability of senescent myoblasts to differentiate to committed cells is weakened, leading to loss of muscle mass and function, which reduces the quality of life of the elderly [54]. On the other hand, senescent cells release some factors to promote the senescence of the body. For example, Chang-Jun Li et al. reported in 2021 that with the onset of senescence, pro-inflammatory and senescence subtypes of immune cells, including macrophages and neutrophils, accumulate and release granular calcains (GCA), which promote bone senescence [55].

There are many ways to characterize aging in general. The detection methods of aging can be based on physiological characteristics, histological characteristics, and biomarkers of aging. Among them, many kinds of physiological characteristics can be detected such as skin, muscle, metabolism, motor, spatial, cognitive, and memory abilities. For the histological characteristics, we can detect them including hypothalamus, kidney, liver and heart. And senescence markers such as p16, lipofuscin, and γH2AX can be detected to characterize aging. First, in the detection of aging based on physiological characteristics, the most intuitive manifestations are the skin state, hair, action, etc. Then, other indicators can also be measured to characterize senescence. For human, we can detect the strength of muscle by grip strength test [56] and distinguish the young and senescent adults by gait indicators [57]. And other physiological measures such as heart rate and blood pressure are also related to senescence, in addition, brain, kidney, cardiovascular, and bone play roles in predicting senescence, and these indicators need to be considered together normally [58]. The AARC-10 SF and AARC-50 cognitive functioning subscale can capture a number of senescence-related changes including physical, psychological, and cognitive aspects [59]. For mice, we can measure respiration and metabolism used by the metabolic cage and through the respiratory exchange ratio (RER) [60] to determine the degree of senescence or used by metabolic treadmill in research [61]. In addition, we can test strength and muscle of mice through small animal handgrip dynamometer [62] or a wire suspension test [63, 64]. For the measurements of bone and fat, we can measure the volume of visceral and subcutaneous fat and analyze the the cortical bone and trabecular bone of the mice by bone imaging [61]. There are differences in motor, coordination, and endurance between young and senescent mice; thus, it is feasible to judge senescence by track test [65] and wood rod test [64]. According to previous studies, mice exhibit higher anxiety like behaviors [66] with senescence. So it is a basis for assessing motor activity and anxiety behavior in mice [67] by the open field test [68] to determine the degree of senescence. Moreover, spatial, cognitive, and memory abilities are also important indicators of senescence [57, 69–71], which can be assessed by fear conditioning [72], Y or T maze [63], the Barnes maze test [68, 73], and Morris water maze experiment [72]. In addition to assessing aging based on the physiological characteristics described above, the status of aging can also be measured based on histological characteristics and biomarkers of aging. Astrocytes and microglia are mediators of innate immune responses in the central nervous system (CNS) that become over-activated with age [73]. It was reported that glial fibrillary acidic protein (GFAP) is a specific marker of astrocytes and IBA1 is a marker of microglia [31]. GFPA is a specific marker of astrocytes, which is a major intermediate filament of astrocytes [74] and contributes to astrocytic differentiation and reactivity [75]. The accumulation of GFAP in astrocytes causes increased apoptosis of astrocytes and their surrounding neurons [75]. IBA1 is an actin-cross-linking protein in microglia [76]. It plays a key role in microglia activity [77]; thus, IBA1 is a specific marker of microglia [78]. The activation status of microglia can be reported by IBA1 immunity, which indicates the role of microglia in the central nervous system, especially in the maintenance of brain homeostasis [79]. Senescent microglia and astrocytes are often detected in the senescent brain [80, 81]. Therefore, senescence can be characterized by examining astrocytes and microglia. In addition to the markers of senescence listed above, the detection of several factors and the proportion of senescent cells in organs can also accurately determine senescence, such as hypothalamus, kidney, liver, and heart. It was found that senescent mice had increased hypothalamic inflammation and increased levels of various saturated fatty acids in the hypothalamus. Meanwhile, hypothalamic nuclei such as arcuate nucleus (ARC), ventromedial hypothalamic nucleus (VMH), and lateral hypothalamic nucleus (LH) also showed higher number of COX-2-positive cells and higher COX-2 immunoreactivity [82]. For kidney, SA-β-gal staining can be used to determine senescence, showing that the proportion of senile positive cells increased significantly with the increase of senescence [67]. γH2AX as a marker of senescence can be detected in mouse kidney to characterize senescence by using immunoblotting [67]. What else, researchers performed histological analysis of the kidney, using hematoxylin and eosin (h&E) staining and Masson's trichrome (MT) staining to observe the renal structure and renal fibrosis. It was found that the positive area of renal fibrosis increased significantly in the elderly group and the levels of serum BUN and Cr, which can be used to assess age-related renal dysfunction, were significantly increased in the elderly group [83]. Also, for the liver, Sabira Mohammed et al. [84] isolated hepatocytes and liver macrophages from senescent mice and found that markers of M1 macrophages, expression of pro-inflammatory factors (TNFα, IL-6, and IL1β), and markers of fibrosis were significantly upregulated in the liver with increasing age. In addition, they demonstrated that immunofluorescence staining of the livers of young and senescent mice and confirmed that the expression of P-MLKL was increased in the liver of senescent mice compared with young mice. Several other markers of senescence have also been used to characterize the senescence of liver, such as p16 [85], lipofuscin, and γH2AX [67, 86], in which we can detect the expression of lipofuscin and γH2AX by SBB staining and western blot separately [67]. Moreover, it is known that the occurrence of cardiovascular diseases is greatly related to senescence, such as cardiac failure [87–89], arrhythmia [90], coronary artery dysfunction [91], atherosclerosis, and myocardial infarction [92]. We can detect amyloid deposition and lipofuscin accumulation to characterize cardiac senescence [90]. Also, it is found that mTOR phosphorylation is detected to increase in the heart of senescent mice [93], which is a key regulator of autophagy [94]. In summary, all research results indicated us that there are many characterization methods to detect senescence, and we can characterize whether the treatment used is effective in treating senescence according to different senescence indicators.

The above is a small summary of the causes of senescence and cellular senescence, and the characterization of senescence in mice (Table 1). Microscopically, stem cell depletion is the root cause of the appearance of senescence [32, 42, 95–97]. Stem cells are divided into embryonic stem cells and adult stem cells according to their developmental stages, and according to the developmental potential of stem cells, they are divided into three categories: totipotent stem cells (TSCs), pluripotent stem cells, and unipotent stem cells. Of interest, delaying cellular senescence or increasing the number of stem cells may greatly delay senescence. In this regard, stem cell-based cell therapies have been proposed for the treatment of senescence. Among them, MSCs as a kind of pluripotent stromal cells that have the potential of unlimited proliferation, differentiation, migration, homing, and immune regulation properties. And MSCs can be derived from various tissues, including bone marrow, bone, umbilical cord, and adipose tissue, of which transplantation has received extensive attention as a more promising treatment method for anti-senescence.

**Table 1:** A variety of methods to characterize the senescence of mice

Characterization of senescence	Methods
Skin	
Hair	
Action	
Respiration and metabolism	Metabolic cage (RER), metabolic treadmill
Strength and muscle	Small animal handgrip dynamometer, wire suspension test
Bone and fat	Measure the volume of visceral and subcutaneous fat, bone imaging
Motor, coordination, and endurance	Track test, wood rod test
Anxiety-like behaviors	Open field test
Spatial, cognitive, and memory abilities	Fear conditioning, Y or T maze, the Barnes maze test, morris water maze
Astrocytes and microglia	GFAP↑ IBA1↑
Hypothalamus	Inflammation↑ various saturated fatty acids↑ COX-2 positive cells↑
Kidney	SA-β-gal positive cells↑ γH2AX↑ BUN↑ Cr↑
Liver	Markers of M1 macrophages↑ pro-inflammatory factors (TNFα, IL-6 and IL1β) ↑ P-MLKL↑ p16↑ lipofuscin↑ γH2AX↑
Heart	Amyloid deposition↑ lipofuscin↑ mTOR phosphorylation↑

↑ = elevated; γH2AX, p16 and lipofuscin are markers of senescence

### MSCs therapy for anti-senescence and senescence-related diseases

MSCs are multipotent cells, which can give rise to mesenchymal and non-mesenchymal tissues in vitro and in vivo and have the properties including self-renewal and multi-directional differentiation capacity. It is well established that MSCs can be obtained from different tissues and organs [98]. Moreover, MSCs should meet at least three criteria: adherent growth, some specific antigens expressed on the cell surface and the ability to differentiate into adipocytes, osteoblasts, and chondrocytes. Many studies have reported that MSCs can delay senescence and treat senescence-related diseases. In addition, the therapeutic effects of MSCs from different age donors and different passages of MSCs are compared; the following content will describe them in detail.

### Therapeutic effects of MSCs on skin, hair growth, and prolonging lifespan

Hair loss and graying are the direct representation of senescence. Currently, alopecia can be treated with drugs and surgery. However, these methods face many challenges; the treatment of drugs is not very effective and has side effects [99]. For surgical treatment, the effect is related to the different surgical skills, and the number of surgical treatments for the same patient cannot be more than three times [100]. Therefore, a more effective treatment for alopecia needs to be developed. According to the literature reviewed, alopecia is related to hair follicle degeneration, and hair follicle stem cells (HFSCs) can promote hair follicle regeneration and hair growth [101, 102]. The hair growth cycle includes the growth phase, the decline phase, and the resting phase [103], and senescent HFSCs increase the resting phase of hair follicles and shorten the growth phase. Nuclear factor of activated T cell cytoplasmic 1(NFATc1) plays an important role in osteoclast differentiation [104]. The accumulation of NFATc1 was shown to prolong the resting phase, eventually leading to hair loss. However, surprisingly, NFATc1 inhibitor was proved that can reactivate the senescent HFSCs and promote hair growth [105]. Therefore, Nfatc1 can coordinate HFSCs to restore their excellent function of hair regeneration. MSCs also play a role in hair regeneration, which depends on the ability of migration, homing, and differentiation. Moreover, MSCs have paracrine effect to release trophic factors, including cytokines and various growth factors, which plays an important role in hair regeneration and repair [99]. Bone marrow-derived MSCs (BMSCs) have been proved that they can promote the proliferation of HFSCs and lead to the transition of hair follicles from the resting phase to the growth phase, thereby promoting hair follicle regeneration and hair growth [101]. Hair follicular dermal papilla cells are key components of hair follicles. Human umbilical cord-derived MSCs (hUC-MSCs) have been proved that have the capacity to protect hair follicular dermal papilla cells to treat alopecia [106]. Others, such as dental-derived MSCs, hair follicle-derived MSCs, and amniotic fluid-derived MSCs, also can accelerate hair regeneration in a similar way [100, 107, 108]. All of these indicate that the various types of MSCs have therapeutic effect on alopecia.

The change of skin status is also one of intuitive manifestations of senescence. The skin is made up of three main layers including the epidermis [109, 110], the dermis [111] and the subcutaneous tissue [112]. According to some studies, it is shown that skin senescence is associated with collagen loss, increased oxidative activity, and increased matrix metalloproteinases. MSCs can secrete factors required for skin regeneration, increase collagen synthesis, and inhibit the expression of mechanical metalloproteinases, which has a good application prospect in skin regeneration [113]. In addition, studies demonstrated that young BMSCs have the potential to regenerate the skin of senescent rats, which mainly utilizes the antioxidant properties of BMSCs to improve oxidative stress in senescent skin of rats and promote regeneration of senescent skin [114]. Additionally, UV radiation is also one of the key factors of skin senescence, which is easy to damage the dermis of skin and destroy fibrous tissue, called light aging. MSCs can promote skin regeneration by increasing cell proliferation and neovascularization, being able to produce collagen and elastin fibers and inhibit the activation of metalloproteinases, in addition to protecting against UV radiation-induced senescence by tissue structural regeneration [113, 115].

Moreover, scientists have conducted numerous experiments to prove that MSCs have an effect on prolonging life span. For example, Li Jun et al. tried transplanting fetal rat MSCs into senescent mice and according to the morphology, mental state and activity of the mice, the changes of heart, spleen, kidney, lung, colon, skin and other tissues, it was proved that fetal mouse MSCs had anti-senescence effects, which may be through differentiation to replace some cells, or may be by improving blood supply pathways or cytokines secreted by stem cells [116]. Amyotrophic lateral sclerosis (ALS) is a neurodegenerative disease; it has been proven that human adipose tissue-derived stem cells (ASCs) can prolong the life span of amyotrophic lateral sclerosis mice [117]. And it was researched that adipose-derived mesenchymal stem cells (ADSCs) treatment can promote mitophagy to improve the characteristics of senescence and demonstrated for the first time that allogeneic stem cell therapy can improve senescence-related signs and phenotypes through mitochondrial quality control. These suggest the importance of stem cells in senescence-related diseases [118]. In the study of aging, we usually use senescent animal models, which are generally divided into natural senescence animal models and accelerated senescence animal models. Moreover, in order to demonstrate the anti-senescence effect of MSCs more quickly and intuitively, researchers have invented accelerated senescence mouse models, which play a significant role in the study of senescence experiments. The therapeutic effects of drugs or MSCs can be verified by characterizing the accelerated senescent mouse. Commonly accelerated senescent mouse models include d-galactose-induced senescence model [119, 120], doxorubicin-induced senescence mice [67, 121], senescence-accelerated mouse/prone (SAMP) [122, 123], total body irradiation (TBI) model [124, 125], ozone-induced senescence model [126, 127], etc. In addition, there are several genetic mutant mice that have been widely studied in premature aging. Klotho mouse is caused by defective expression of klotho gene, which resulting in a syndrome similar to human aging [128]. Bmi-1^−/−^mouse is an animal model of premature aging, showing retarded growth, decreased cell proliferation, increased apoptosis, and premature aging [129]. Xpg^−/−^mouse is a model of accelerated senescence; it is characterized by shortened lifespan, significant neuropathy, and other senescence-related abnormalities [130]. Werner syndrome is a rare autosomal recessive disorder characterized by accelerated aging [131, 132], so the mouse model of Werner Syndrome is a model of aging [133]. Ercc1^−/Δ^ mice represents an accelerated model of aging-related peripheral neuropathy, which can help to discover the molecular mechanism of peripheral nerve degeneration and screen out treatments to prevent, delay, or reverse peripheral neuropathy [134]. Moreover, drosophila and Caenorhabditis elegans are also recognized animal models for senescence studies [131, 135]. For instance, drosophila model of Werner syndrome exhibits physiological signs of aging, such as shortened lifespan, increased incidence of tumors, muscle degeneration, and behavioral changes [136]. Numerous senescence models are helpful to reveal the mechanisms of aging, achieve the purposes of anti-aging, and treat senescence-related diseases. A study reported that mesenchymal progenitor cells (MPCs) were implanted into accelerated senescence mouse model; it was observed that the transplanted mice had improved cortical bone mass and alleviated osteoporosis compared with the control group. And the average life span was about 30% longer than that of nontransplantation control group [137]. In addition, Bmi1-deficient mice is an accelerated senescence mouse model, and it is confirmed that the premature senility phenotype can be ameliorated after the transplantation of amniotic membrane mesenchymal stem cells (AMSCs) [129].

Stem cells have also been shown to be effective in clinical treatment. It was verified that repeated intravenous administrations of autologous ADSCs improved the symptoms of ALS patients without any adverse effects [138]. Hutchinson–Gilford progeria syndrome (HGPS) is a rare and fatal genetic disorder of accelerated senescence in children that shows symptoms of senescence at an early stage, resulting in slow growth, hair loss, and a heightened voice [139]. It was reported that a case of HGPS patients showed significant growth in weight and height after receiving allogeneic haploidentical transplants of adipose SVF containing MSCs, along with about 50% increase in IGF-1, which may prolong the life of HGPS patients [140]. Senescence refers to the gradual physical degeneration of the body with the increase of age. Similar to senescence, frailty is the result of multilevel degradation of interacting physiological systems, which is characterized by decreased physiological reserve and decreased resistance to stress, involving physiological changes in the neuromuscular system, metabolism, and immune system. Such changes increase the risk of disability, delirium, falls, and even death in the elderly [141]. Frailty is the main phenotype of accelerated senescence, which describes multiple organ dysfunction or multiple diseases in the elderly, as well as increased susceptibility to other diseases [142]. The main clinical features of frailty are reduced muscle mass, anorexia, weight loss, and decreased energy expenditure [143]. Chronic inflammatory response can accelerate the occurrence and development of frailty syndrome. IL-6, CRP, TNFα, and CXCL-10 are inflammatory factors; the levels are elevated during frailty. Therefore, they can serve as inflammatory markers related to frailty syndrome [142, 143]. There was a randomized and double-blind study of allogeneic human mesenchymal stem cells (allo-hMSCs) transplanted into frail patients compared with placebo. After several months, the two frailty syndrome measures, including the physical fitness measure and the validation marker, were found to improve [144]. Moreover, allo-hMSCs had been shown to reduce frailty, improve cardiovascular function, reduce inflammation, prolong life span, and improve quality of life in frail patients [95], and it was confirmed that intravenous injection of allo-hMSCs had great safety, which provided support for the treatment of frail elderly people [145]. These studies indicate that MSCs have great potential in delaying senescence, and young MSCs seem to have a better therapeutic effect than senescent MSCs.

### Therapeutic effects of MSCs on osteoporosis

We all known that osteoporosis becomes common as aging occurs. Osteoporosis is a progressive systemic bone disease associated with age, which is characterized by low bone mass and large bone fragility [46]. In osteoporosis patients, MSCs have a reduced ability to differentiate into osteoblasts and an increased ability to differentiate into adipocytes, resulting in reduced bone formation [146]. In particular, most elderly patients have substantially increased morbidity and mortality [147, 148]. Although there are some drugs for the treatment of osteoporosis, these drugs may have adverse reactions and these treatments are not completely effective for all patients [46]. Therefore, more effective treatments for osteoporosis are needed. As is known that MSCs not only have the ability to differentiate into adipocytes, osteoblasts, and chondrocytes [148, 149], but also secrete factors involved in bone repair [46]. So, MSCs can be used as an alternative therapy for age-related osteoporosis. There are two approaches to MSCs transplantation for the treatment of osteoporosis, including systemic and local transplantation. Both methods have certain drawbacks, local transplantation can lead to poor cell survival, while systemic transplantation can cause cells to accumulate in the lung or in areas of inflammation. To address these defects, the transplanted MSCs can be genetically modified to improve the therapeutic efficacy of osteoporosis, such as activating cytokines and transcription factors [150, 151]. At present, a large number of studies have demonstrated the potential role of MSCs from various sources in the treatment of osteoporosis. hUC-MSCs have been shown to have broad application prospects in the treatment of osteoporosis. UC-MSCs were loaded into a biomimetic artificial bone scaffold material, and then, the bone scaffold material was implanted into BALB/c nude mice subcutaneously. The results showed that UC-MSCs could effectively induce bone formation [147, 152]. SAMP6 mouse is an accelerated senescence mouse model that develops osteoporosis at an early stage. A study showed that normal allogeneic mouse BMSCs were locally injected into SAMP6 mice, and it was found that such MSCs could increase trabecular bone and bone remodeling, alleviate bone mineral density loss, and prevent osteoporosis [46, 153]. It was reported that BMSCs transplantation could not only alleviate the loss of bone mineral density (BMD) in the knee joint of aged recipient mice, but also increase the trabecular meshwork and BMD of the mice, which proved that BMSCs could effectively restore bone structure and BMD in senescent mice. In addition, this study demonstrated that aged mice transplanted with BMSCs had a longer life span compared with the control group, which reflecting the link between MSCs and aging [154]. In addition, Maf as a factor has been shown to regulate and promote osteogenic differentiation of MSCs [155]. Therefore, it is possible that Maf may have the ability to assist MSCs in the treatment of osteoporosis.

In addition, there was also a clinical study on the treatment of osteoporosis. It was a randomized, open-label and phase I/IIa study, which conducted by twenty subjects with osteoporotic vertebral compression fractures (OVCFs) that were randomly assigned to three groups to confirm the feasibility, safety, and effectiveness of Wharton's jelly-derived mesenchymal stem cells (WJ-MSCs) and teriparatide in OVCFs. Although there were some possible MSC-related complications such as pulmonary embolism and tumor formation during the study, the result showed that their combination therapy was tolerable and feasible, which could help to promote bone healing in clinical [156]. Collectively, these clinical studies indicate that MSCs are promising for the treatment of senescence-related osteoporosis.

### Therapeutic effects of MSCs on CVDs

It has been mentioned before that senescence can lead to the occurrence of CVDs, such as myocardial infarction (MI), heart failure, and arteriosclerosis. It was reported that the transplantation of multipotent germline stem cells (mGSCs) derived from neonatal mouse tests into the hearts of mice with ischemic heart failure can improve cardiac function by promoting angiogenesis and delaying cellular senescence [157]. It was proven that embryonic stem cells (ESs) can be induced to differentiate into spontaneously beating cardiomyocytes in vitro [158]. Based on this characteristic, in 2020, researchers developed a method to generate cardiac organoids that were very similar to internal organs by using mouse embryonic stem cell-derived embryoid bodies under the condition of FGF4 and extracellular matrix [159], which was of great interest for the treatment of CVDs. In short, ES has been shown to alleviate heart failure models [160] and regenerate the cardiovascular system [161]. MSCs are more potential cells for treatment, it has been reported that it can repair infarcted myocardium, and pretreatment of aged MSCs under glucose depletion conditions has been shown to improve cardiac function after MI. In addition, it was observed that the potential of MSCs to repair senescent infarcted myocardium decreased with age [162]. Autologous Ad-MSCs were determined that they can significantly improve the levels of HDL, LDL, and residual particle (RLP) cholesterol, which were safe and effective in the treatment of arteriosclerosis [163]. In order to further explore the mechanism of stem cell therapy for CVDs, Rosalinda Madonna et al. designed an experiment and demonstrated that active microcarriers (PAM), which can release vascular endothelial growth factor (VEGF) combined with adipose tissue mesenchymal stromal cells (AT-MSCs), can exert paracrine effects, inhibit cell apoptosis, reduce fibrosis, increase arterial generation, and improve myocardial shortening, which may have therapeutic effects [164].

Analysis of the reported trials of MSCs in the clinical treatment of acute myocardial infarction (AMI) showed that MSCs therapy was associated with a significant improvement in left ventricular ejection fraction (LVEF) increased by 2.62% in patients transplanted with 10^7^–10^8^ MSCs, and the effect was maintained for up to 24 months. In addition, there were no adverse events caused by MSCs treatment found [165]. Moreover, MSCs as regenerative treatments for heart failure were determined to be safe and effective. Due to the self-renewal differentiation capacity and immunomodulatory properties, MSCs transplantation significantly improved LVEF and reduced left ventricular end-systolic volume (LVESV) and left ventricular end-diastolic volume (LVEDV). In different origin, the therapeutic effect of UC-MSCs seemed to be better than BM-MSCs, and the injection dose of (1–10) × 10^8^ cells had a better therapeutic effect [166].

**Fig. 2:**
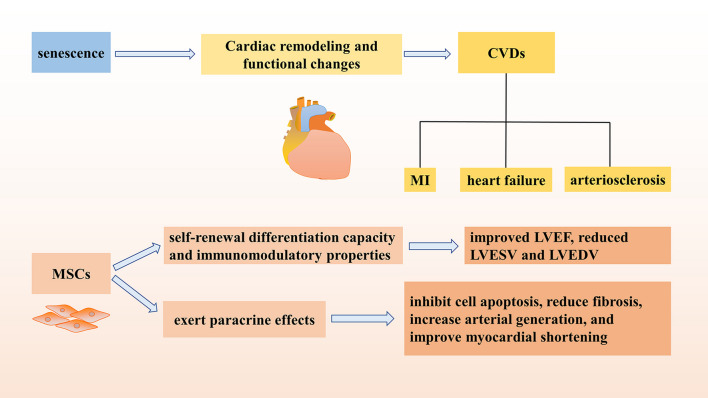
Senescence contributes to CVDs and MSCs have the ability to improve the treatment of CVDs

Taken together, MSCs transplantation can ameliorate a variety of CVDs by increasing angiogenesis, reducing fibrosis, reducing infarct size, improving myocardial shortening, and exerting paracrine effects, which has great potential for ameliorating senescence (Fig. 2).

### Therapeutic effects of MSCs on neurodegenerative diseases

**Fig. 3:**
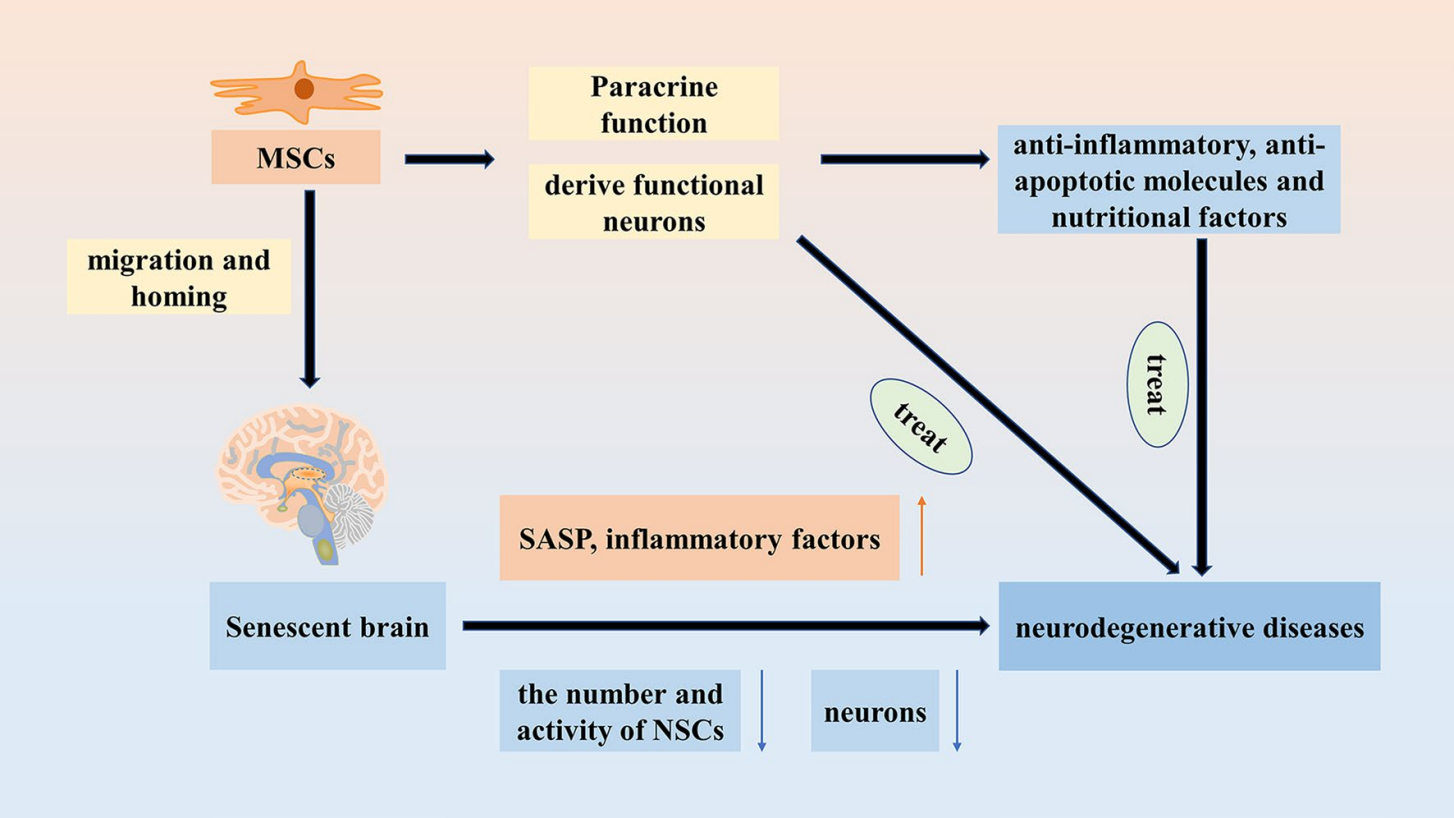
Senescence is a major risk factor for neurodegenerative diseases. MSCs have the ability of migration and homing to make them play a role in the aging brain, and they can treat neurodegenerative diseases by deriving functional neurons and the paracrine function

Senescence is a major risk factor for neurodegenerative diseases. And the secretion of inflammatory factors increases because of the SASP. The increased inflammation is associated with the generation of senescence and neurodegenerative diseases [167]. With the onset of senescence, PD and AD increase, which may be related to the decrease in the number and activity of neural stem cells (NSCs). NSCs can generate new neurons [168] to improve the function of CNS. In the study of NSCs, nicotinamide adenine dinucleotide (NAD^+^) was found to not only maintain healthy mitochondria, but also protect NSCs, muscle stem cells (MuSCs), and melanocyte stem cells (McSCs) from senescence. Nicotinamide riboside (NR) as a NAD^+^ precursor was proved to delay the senescence of NSCs and McSCs as well as prolonging the life span of mice [169]. Therefore, the supplementation of NAD^+^ contributes to the effects of NSCs. As mentioned above, the hippocampus is associated with learning and memory. In addition, the decrease in the number and maturity of neurons in the hippocampus are also closely related to neurodegenerative diseases [170]. MSCs can derive functional neurons such as dopaminergic neurons [171–173], which makes MSCs have greater prospects in the treatment of neurodegenerative diseases. In addition, transplanted MSCs have the potential of migration and homing [174, 175], which make MSCs play therapeutic role at particular sites. Moreover, MSCs have the ability to secret some anti-inflammatory, anti-apoptotic molecules, and nutritional factors [176–178]. All of the characteristics of MSCs increase neuroprotection and make them have the function to improve neurodegenerative diseases. Therefore, MSCs are effective cell therapies for the treatment of brain deterioration (Fig. 3).

Based on these studies, researchers tried to apply MSCs to clinical studies. Kim et al. [179] conducted a phase I clinical trial in nine patients with mild-to-moderate AD, and three repeated injections of MSCs were performed (4-week intervals) in all nine patients. While showing a certain therapeutic effect, it was found that the patients had an adverse symptom of fever [179]. To investigate the subject further, their team continued their research and showed that the symptom of fever was due to the increased level of pro-inflammatory cytokines in the cerebrospinal fluid of AD patients injected with hUC-MSCs. However, the specific cause of inflammation after MSCs transplantation had not been clearly pointed out, which required a breakthrough in future studies and solutions to reduce fever and inflammation after MSCs transplantation to improve the effect of MSCs in the treatment of AD [180]. Moreover, other clinical trials researched that MSCs had some therapeutic effects on ALS [181] and PD [182]. In conclusion, two independent reviewers conducted a meta-analysis of 71 clinical trials on cell therapy for neurodegenerative diseases, assessing the safety and efficacy of regenerative cell-based therapies in neurodegenerative diseases. Most reports proved that the application of regenerative cell-based therapies in patients was safe and feasible, and more extensive researches on different treatment strategies and dosing regimens are needed in the future [183].

### Therapeutic effects of MSCs on premature ovarian failure (POF)

POF is a common endocrine disease, which can lead to female infertility. The causes of POF may be related to genetic defects, autoimmunity, and chemotherapy damage. However, the pathogenesis of POF remains unclear. POF model can be induced with d-galactose, and in the process of senescence, the advanced glycation end products and ROS increase, then gradually impair the ovarian function, and result in POF [184]. Thus, it can be seen that senescence is associated with POF, with increasing age, and most of the ovarian follicles deplete and ovarian function damage, resulting in POF [185, 186]. At present, the commonly used hormone replacement therapy cannot restore the function of the ovary. However, stem cell therapy brings hope for the treatment of POF [187]. hUC-MSCs are an easily accessible type of MSCs, which has been showed that can repair chemotherapy-induced premature ovarian failure [188]. Autocrosslinked hyaluronic acid (HA) was reported that it not only prolonged the retention of UC‑MSCs in the ovary but also boosted the secretory function of UC-MSCs, which can promote follicular survival by activating the PI3K‑AKT pathway. This will be of great help to the clinical application of MSCs in the treatment of ovarian diseases [189]. In addition, human embryonic stem cells (HESC-MSCs) [190] and BMSCs [191] also had been shown to improve POF. These reports indicated that the therapeutic effect of MSCs may be attributed to immunoregulation, which increased the release of anti-inflammatory factors and inhibited the production of pro-inflammatory factors.

In one study, the transplantation of UC-MSCs on collagen scaffold was documented for the first time. Patients were randomly divided into two groups: one group received UC-MSCs transplantation, while the other group received UC-MSCs combined with collagen transplantation. UC-MSCs or collagen/UC-MSCs were injected into the ovary of patients, and each patient was followed up for at least 1 year after the first transplantation. The results demonstrated that UC-MSCs partially improved the activation and growth of follicle, and UC-MSCs on collagen scaffold contributed to the long-term recovery of ovarian function as well as improved fertility in POF patients [192]. All of these studies demonstrated the promising effects of MSCs in the treatment of POF.

### Therapeutic effects of MSCs on premature aging disorders

Premature aging disorders are rare human disorders characterized by accelerated aging. Some of them are caused by mutations in genes encoding DNA repair proteins, such as Werner syndrome (WS), Bloom syndrome, and Cockayne syndrome. Others are caused by mutations in genes encoding A-type laminas or lamina processing enzymes, such as HGPS and restrictive dermopathy. WS and HGPS are the two most widely studied human progeria disorders so far. These two diseases have been the focus of researches in the field of aging in recent years, because the clinical characteristics of patients are similar to physiological aging, including alopecia, hair graying, growth retardation, osteoporosis, cataract, hearing loss, atherosclerosis, cardiovascular diseases, and early malignant tumors [193, 194]. The pathogenesis of premature aging is mainly related to genomic instability, telomere attrition, loss of protein homeostasis, mitochondrial dysfunction, dysregulated nutrient perception, stem cell failure, cell senescence, and altered intercellular communication [193]. Firstly, WS is an adult-onset progeria syndrome caused by an autosomal recessive mutation, and patients present with premature aging symptoms such as gray hair, alopecia, skin atrophy, ulcers, and retardation of growth in their twenties [195]. In addition, WS patients also have a high incidence of cancer, and they are prone to soft tissue sarcoma and osteosarcoma. The most common causes of death are cancer and myocardial infarction [196]. It was proven that WS was a stem cell dysfunction-associated disease, and significant senescence was observed in MSCs [197]. In a study, the NRF2 gene in the human embryonic stem cell model of WS was reprogrammed to obtain hMSCs with enhanced self-renewal and stress resistance. They had the abilities to delay cell senescence, improve engraftment efficiency and regeneration in vivo, and had better anticancer effects, which provided a good prospect for the treatment of WS and other premature aging disorders [198]. Secondly, HGPS is caused by LMNA gene mutations, which is strikingly similar to the normal aging process and cannot be diagnosed at birth, but prominent symptoms can be observed after 2 years of age. With the increase of age, the skin of patients becomes atrophic, and the main cause of death is cardiovascular disease [199]. The shortened life span and altered stem cells play important roles in the pathogenesis of premature aging syndromes. Therefore, the timely renewal of senescent or dysfunctional stem cells is required to protect patients [200]. As previously mentioned, after transplantation for MSCs, the weight and height of the patients increased significantly, and the patients may have a longer life [140].

In conclusion, MSCs have a wide application prospect in human aging disorders and a large number of studies are needed to conduct better treat premature aging disorders in the future.

### Therapeutic effects of MSCs on chronic kidney disease (CKD)

CKD is mainly caused by inflammation, oxidative stress, and premature aging, leading to decreased quality of life, poor health, and premature death [201, 202]. Therefore, we can focus on inflammation and premature aging to treat CKD. Although drugs and surgical treatment have certain effects, they cannot regenerate and restore the function of tissue, so better treatments are needed. The pluripotency and paracrine mechanism of MSCs make them have great advantages in the treatment of CKD by the recovery of tissue damage and the suppression of inflammation [203, 204].

Diabetic nephropathy is one of the most serious complication of diabetes mellitus and the main cause of end-stage chronic kidney disease. A study demonstrated that UC-MSCs were injected into STZ-induced DN rats via the tail vein. Two weeks later, the researchers measured the blood glucose, renal function, and cytokines in the kidney and blood. The results suggested the improvement of renal function and showed that these functional parameters were significantly improved and the pro-inflammatory and pro-fibrotic factors were also significantly reduced, which may be related to the inhibitory effects of UC-MSCs on inflammation and fibrosis [205]. Camel Wharton jelly mesenchymal stem cells (CWJ-MSCs) were proved to have great potential in the treatment of canine models of CKD [206]. It was performed by injecting CWJ-MSCs into the kidneys of 5/6 nephrectomy dogs under ultrasound guidance, and the results showed a significant reduction in both serum urea and creatinine levels, as well as a decrease in NGAL, KIM-1 gene expression and an increase in VEGF, EGF gene expression, suggesting renal tissue repair. The study suggested that WJ-MSCs had good efficacy in canine CKD models [206].

Moreover, due to the risk of sensitization of allogeneic MSCs transplantation, autologous MSCs transplantation seems to be more advantageous [207]. The first double-blind, placebo-controlled trial of allogeneic BMSCs in thirty patients with type 2 diabetic nephropathy was used to evaluate the safety of MSCs infusion, and although the trial showed low efficacy, no adverse events related to the infusion were noted during the 60-week study [208]. There was another single-arm study that seven patients with CKD caused by different etiologies were intravenously injected with autologous MSCs and followed up for 18 months. The final results showed that a single dose infusion of autologous MSCs was safe and well tolerated in patients with CKD [209]. In addition, many other clinical trials had confirmed the efficacy and safety of MSCs in the treatment of CKD [203]. Although MSCs have a promising efficacy in the treatment of CKD, their efficacy in the treatment of severe kidney disease is limited by the low survival rate [203, 210]. In order to increase the survival rate of MSCs to improve the function and therapeutic effects, MSCs can be pretreated. There are various pretreatment methods, including the incubation of cytokines or compounds, such as DHA, SNP, DPO, atorvastatin, melatonin, and the application of some supporting materials, such as thermosensitive hydrogel, chitosan-based hydrogel, fucoidan [203, 211]. In summary, various studies have shown that MSCs have promising safety and immunosuppressive effects in the treatment of CKD.

### Therapeutic effects of MSCs on chronic obstructive pulmonary disease (COPD)

COPD is associated with systemic inflammation [212], which accelerates with age [213, 214]. Most patients with COPD are accompanied by chronic bronchitis and emphysema [215]. COPD can reduce the quality of life and shorten the life span of patients. Current treatments can only relieve symptoms but cannot fundamentally cure the disease. Recently, the paracrine and immunomodulatory effects of MSCs make them have great application prospects in the treatment of COPD [215]. WJ-MSCs showed pulmonary regenerative effects in the COPD mouse model [212]. Daniel Weiss et al. conducted a study of sixty-two patients with moderate-to-severe COPD who received intravenous infusion of allo-hMSCs or placebo. The patients received four infusions per month and were followed up for 2 years after the first infusion, and no significant adverse reactions were observed. In addition, MSCs can reduce the inflammation caused by COPD, which provide a clinical basis for the treatment of COPD [216]. A phase I, prospective, patient-blinded, randomized, placebo-controlled design was that placebo and unidirectional endobronchial valves in combination with MSCs were injected into 10 patients with severe emphysema. The results demonstrated the safety of the combination in the treatment of severe emphysema, and the treatment reduced systemic inflammation and improved lung function in patients with severe COPD [217]. UC-MSCs had also been shown to have therapeutic effects on COPD. There was a study that allogeneic UC-MSCs were injected into twenty patients with COPD, followed up for 6 months after the first infusion [218]. Then, they evaluated safety, pulmonary function tests, and quality-of-life indicators. No adverse events related to UC-MSCs administration were found; however, C-reactive protein (CRP) and 6MWT values were not significantly decreased after treatment. In conclusion, the study demonstrated that UC-MSC therapy was safe and could improve the lives of patients with moderate-to-severe COPD [218].

With more and more researches on COPD, a study explained the effect of MSC infusions on lung function in COPD patients with high CRP levels [219]. Based on a study they previously reported, MSCs had a good safety profile but no functional efficacy was observed in the treatment of COPD [216]. The researchers conducted a post hoc analysis with stratification based on levels of CRP to determine the effects of MSCs administration in COPD patients with varying circulating CRP levels, with 4 monthly infusions of bone marrow-derived allogeneic MSCs and placebo. The results showed decrease of circulating CRP in patients treated with MSCs and improvement in lung and global functions of patients. In addition, no obvious adverse reactions were found during the observation period of 2 years [219]. Numerous studies have demonstrated the role and safety of MSCs from different sources in the treatment of COPD [215, 216, 220]. However, the long-term safety and efficacy of the treatment need to be verified by a large number of studies.

### Therapeutic effects of MSCs on atherosclerosis

Atherosclerosis is a group of senile diseases, which is associated with age and premature biological aging. Moreover, there is a growing evidence that organismal and cellular senescence promote atherosclerosis [221], induced by lipid deposition and inflammation [222–224]. Atherosclerosis usually causes to the occurrence of CVDs, which has a great threat to human health. The vascular damage repair and inflammatory inhibition effects of MSCs make them widely used in the treatment of atherosclerotic diseases [222, 225–227]. Atherosclerosis is a vascular complication of diabetes. An experiment was conducted based on a diabetic model of rats as well as a cellular model of human endothelial cells. Researchers have investigated the effect of hUC-MSCs on diabetic endothelial cell injury, and the results showed that hUC-MSCs could not only improve blood glucose, but also protect vascular endothelial injury, which had a good therapeutic prospect [224]. Amniotic membrane mesenchymal stem cells (AMSCs), a kind of MSCs with immunomodulatory effect, have been proven to have therapeutic implications for early atherosclerotic plaque formation in apolipoprotein E-knockout mice by modulating the function of macrophage to reduce immune response. It was detected that the pro-inflammatory cytokine tumor necrosis factor α and macrophages were decreased and interleukin-10 (IL-10) was increased in the AMSCs treatment group [228]. Another study demonstrated the effect of MSCs on atherosclerosis. In this study, skin-derived MSCs were injected into apo E^−/−^ mice. The research showed an increased release of the anti-inflammatory cytokine IL-10, and a decreased release of inflammatory cytokines TNF-a and IL-1b, thereby exerting immunosuppressive effects to ameliorate atherosclerosis in mice [229].

In addition to inhibiting inflammation and repairing vascular damage, MSCs can treat atherosclerosis by reducing platelet activation, restoring endothelial function [226]. In a study, MSCs isolated from human term placenta were proven that they could express prothrombotic and antithrombotic proteins, reduce CD36-mediated platelet activation induced by oxidized LDL to treat atherosclerosis [230]. In addition, the restoration of endothelial function by MSCs also contributes to the improvement of atherosclerosis. MSCs were shown to improve endothelial function and plaque formation in high-fat diet-induced apoE^−/−^ mice. And it was verified that MSCs exerted their therapeutic effects through paracrine action rather than differentiation [231].

In conclusion, MSCs-based therapy is an effective strategy for the treatment of atherosclerosis. In the future, if MSCs can be induced to differentiate into non-inflammatory cells or vascular endothelial cells in vivo, it will be better for the treatment of atherosclerosis [232].

Taken together, numerous studies demonstrate that MSCs therapy has huge potential in delaying senescence as well as treating diseases related to senescence (Table 2). The mechanism may be due to the immunomodulatory effect of MSCs, which exerts its effect by the capacity of proliferation and differentiation, secreting anti-inflammatory factors and inhibiting pro-inflammatory factors. Also noteworthy is that the young MSCs seem to have better effect in contrast to senescent MSCs. Thus, it is better to choose young MSCs as long as there are sufficient cells for transplantation. Then, we discuss the different stages of MSCs at the cellular level.

**Table 2:** MSCs therapy for senescence-related diseases in clinical trials

Senescence-related disease		Stem cells	Trial size	Treatment measures	Follow-up period	Results
Senescence	Frail elderly patients	Allo-hMSCs	30	Intravenous injection of allo-hMSCs	12 months	Two frailty syndrome measures were found to improve, and it showed reduce frailty, improve cardiovascular function, reduce inflammation, prolong life span and improve quality of life in frail patients, which has great safety [144]
Osteoporosis	OVCF	WJ-MSCs	20	Transplantation of WJ-MSCs and teriparatide	12 months	Promote bone healing, possible MSC-related complications such as pulmonary embolism and tumor formation occurred [156]
CVDs	AMI	BMSCs	45	BMSCs were administered into infarct-related artery	12 months	Slight improvement of myocardial perfusion in the BMSCs group [233]
	Severe ischemic heart failure	MSCs	55	Intra-myocardial injections of MSCs	6 months	Improved myocardial function and no side effects were identified [234]
	Non-ischemic cardiomyopathy	Allogeneic MSCs	22	Intravenous allogeneic MSCs	90 days	Therapy was safe, improved health status and functional capacity [235]
	Heart failure	UC-MSCs	30	Intravenous infusion of UC-MSCs	12 months	Improvements in left ventricular function, functional status, and quality of life [236]
Neurodegenerative diseases	AD	MSCs	9	Repeated intracerebroventricular injections of MSCs	36 months	While showing a certain therapeutic effect, patient had an adverse symptom of fever [179]
	ALS	BMSCs	26	BMSCs via lumbar puncture into the cerebrospinal fluid	18 months	30% of the patients experienced a mild-to-moderate headache, no suspected serious adverse reactions (SUSAR) were observed, slow down progression of ALS [181]
	PD	BMSCs	7	The BMSCs were transplanted into the sublateral ventricular zone by stereotaxic surgery	10–36 months	No serious adverse events occurred but the effectiveness of the treatment was not demonstrated [182]
POF		UC-MSCs	14	Transplantation of UC-MSCs on collagen scaffold	At least 1 year	Partially improved the activation and growth of follicle and improved fertility [192]
CKD	Patients with type 2 diabetic nephropathy	BMSCs	30	BMSCs infusion	60 weeks	Showed low efficacy, no adverse events related to the infusion were noted [208]
		MSCs	7	Intravenous injection	18 months	Showed that a single dose infusion of autologous MSCs was safe and well tolerated in patients with CKD [209]
COPD		Allo-hMSCs	62	Intravenous injection	2 years	Reduced inflammation and no significant adverse [216]
		UC-MSCs	20		6 months	UC-MSC therapy was safe and could improve the lives of patients with moderate-to-severe COPD [218]

## Young MSCs enhance the activity of senescent MSCs

**Fig. 4:**
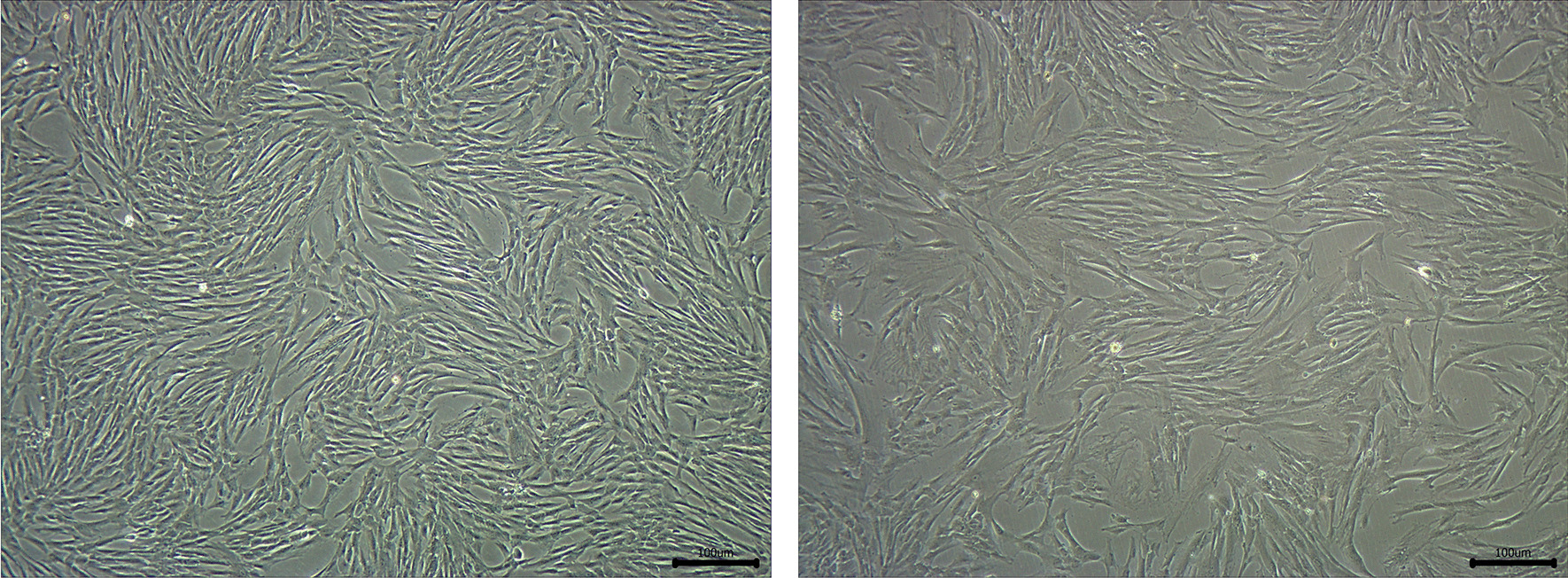
The morphology of hUC-MSCs: young hUC-MSCs at passage 5 (left) and senescent hUC-MSCs at passage 10 (right)

It has been proven that with the senescence of hUC-MSCs, the morphology of the cells changes from elongated fusiform to large and flat (Fig. 4), and the ability of secreting factors also decreases with senescence, such as growth factors, cell adhesion and anti-inflammatory factors, manifested as decreased immunomodulatory capacity [237]. Interestingly, Jinhui et al. discovered that mice injected with young BMSCs had a longer lifespan compared with mice injected with senescent BMSCs [154]. Another study was shown to have a similar phenomenon. MSCs from senescent and young male donors were transplanted into senescent female mice, and they found that the transplantation of young MSCs significantly slowed the loss of bone mineral density, surprisingly, in addition to extending the life span of senescent mice [154, 238]. Therefore, it is better to transplant young MSCs rather than senescent MSCs.

There is evidence for co-culturing MSCs to enhance the activity of other cells. Previously, it was demonstrated that co-culture with MSCs can increase the proliferation and maintenance of hematopoietic progenitor cells, especially co-culture with early passage MSCs [239]. Many researchers have discovered that mesenchymal stromal cell-conditioned medium (MSC-CM) has the similar effect. MSC-CM can ameliorate fibroblast senescence induced by high glucose (HG), and MSC-CM holds promise as an alternative therapy for chronic wounds [240]. Mohadese Hashem Boroojerdi employed microarray assay to analyze the HSCs co-cultured with hUC-MSCs. And they reported that the cell death of HSCs reduced without disturbing the undifferentiated state of HSCs [241]. Moreover, Wang Lixue et al. reported that extracellular vesicle (EVs) derived from hUC-MSCs can promote proliferation and migration of dermal fibroblasts, increase elastic fibers, collagen expression, and reduce the production of matrix metalloproteinase-1 (MMP-1) and matrix metalloproteinase-3 (MMP-3) [115].

**Fig. 5:**
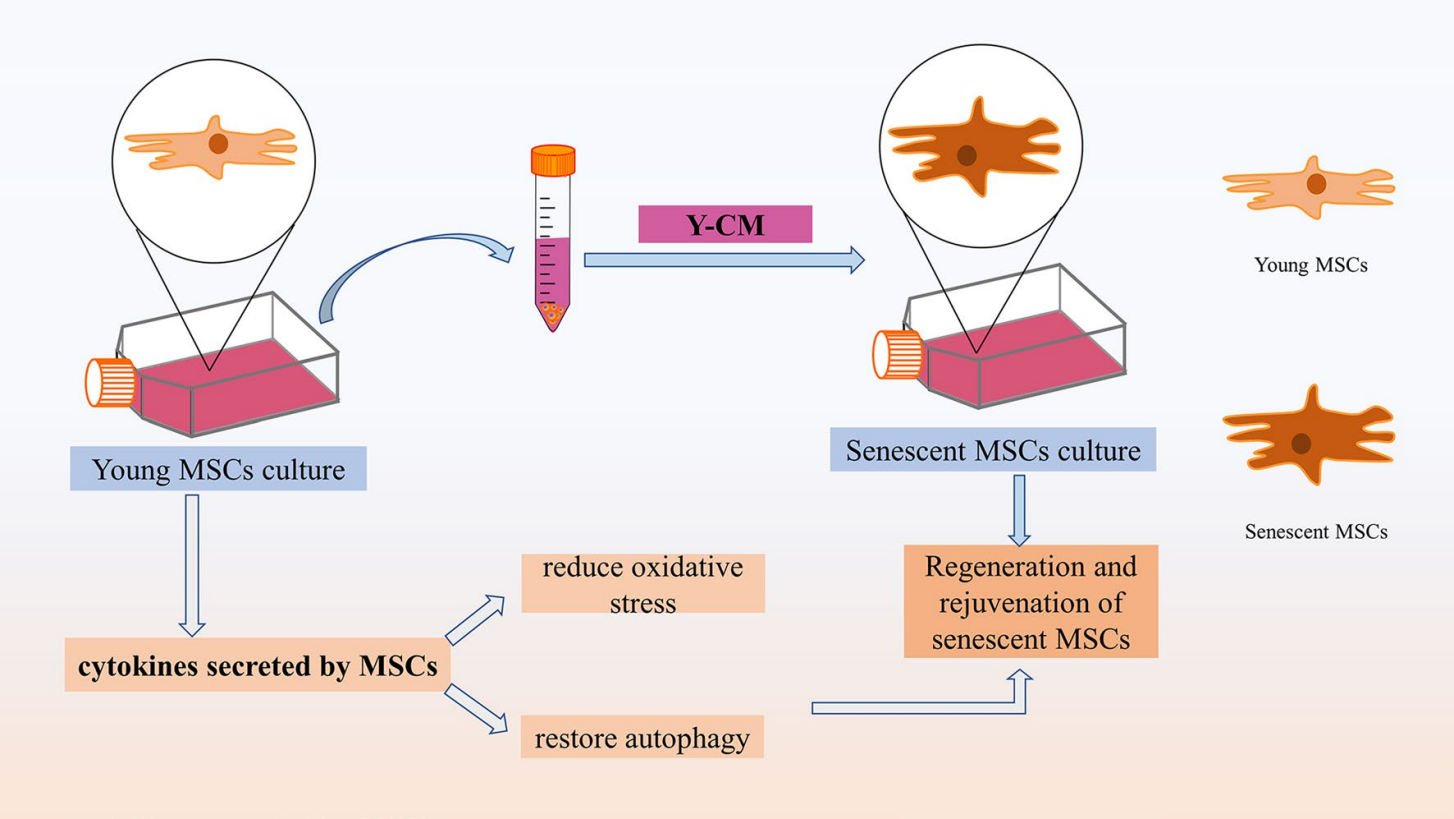
Senescent MSCs were cultured with the CM derived from young MSCs (Y-CM); the senescent stem cells could be rejuvenated by reducing oxidative stress and restoring autophagy

As we known, compared with young MSCs, senescent MSCs exhibit increased cellular senescence and decreased activity [242]. To enhance the activity of senescent MSCs, some researchers tried to stimulate senescent MSCs with exosomes derived from young UC-MSCs, and they found the activity of senescent MSCs enhanced, accompanied by the reduced activity of SA-β-gal and expression of senescence-related factors such as p53, p21, and p16. In addition, treated senescent MSCs were transplanted in the mouse model of MI to test the role, and then, they found and treated senescent MSCs to enhance the function of myocardial repair. And their data suggested that exosomes from young MSCs can improve activities of senescent MSCs and enhance their function for myocardial repair by transferring miR-136 and downregulating Apaf1 [243]. Similar to this study, Madhurima Das et al. treated senescent MSCs with the CM derived from young MSCs (Y-CM) and analyzed the expression of phenotypic markers CD90 and CD45 by flow cytometry, which proved that the senescent stem cells could be rejuvenated [244]. The effect of CM derived from MSCs may have great relation to the role of cytokines secreted by MSCs. According to these studies, it is concluded that co-culture of young MSCs or CM derived from young MSCs can restore the viability of senescent MSCs, which may due to the ability of young MSCs such as reducing oxidative stress and restoring autophagy to rejuvenate senescent MSCs [244] (Fig. 5). Therefore, young MSCs can restore the activity of senescent MSCs, and the therapeutic effect of young MSCs may be better, so young MSCs should be selected as far as possible for clinical application.

## The challenge of MSCs for senescence therapy in clinic

At present, the treatments of a variety of diseases based on MSCs, such as heart, bone, neurodegenerative diseases, and immune diseases, have reached phase I and phase II clinical trials [245]. However, although MSCs have great potential in the treatment of many diseases and senescence, the current clinical treatment status of MSCs shows that MSCs still cannot be applied to clinical treatment on a large scale. The clinical application of MSCs is still at an early stage, and extensive clinical trials are needed to verify [246] the safety and efficacy of MSCs [247]. For efficacy of MSCs, on the one hand, we know that the proliferation and differentiation ability of senescent MSCs are significantly decreased, and thus, it cannot meet the large-scale dose of MSCs required for clinical application. On the other hand, most MSCs are trapped in the lung after intravenous infusion, and this kind of lung first-pass effect causes that a small number of MSCs can be detected in the target organ, thus requiring multiple infusions at the time of treatment to improve the rate of MSCs transplantation [151]. It is known that the pretreatment of MSCs may help to improve the survival rate after transplantation and play a therapeutic role [248]. For example, MSCs need to be subjected to brief oxidative stress before injection, usually treated at 20% O_2_ for 48 h [249]. This kind of brief oxidative stress can improve the survival rate of MSCs after transplantation. Therefore, for the clinical application of MSCs in the treatment of senescence, a large number of trials are still needed to determine the optimal and standard dose of transplantation to maximize the efficacy of MSCs [250].

In addition to the above conditions that affect the therapeutic effect of MSCs, the heterogeneity of MSCs also has an impact on the efficacy. MSCs include variants from donors, tissues, subpopulations, and individual cells, termed heterogeneous [251]. MSCs are present in all organs and tissues, and their heterogeneity is related to many factors, showing heterogeneity at multiple levels, such as donors and tissue sources, cell isolation techniques, culture conditions, and preservation conditions [252, 253]. In conclusion, MSCs are heterogeneous cell population [254]. MSCs derived from different age, health status, gender, gene donors, and tissues show different characteristics. Even of MSCs are obtained from the same individual but at the different sampling location, the properties of the obtained MSCs may be different. A meaningful study confirmed it in 2021 [255]. In this work, they conducted a number of experiments to investigate the different biological characteristics and heterogeneity among different donors of chorionic plate mesenchymal stem cells (CP-MSCs), AMSCs, and decidual plate mesenchymal stem cells (DP-MSCs) isolated from human placenta. CP-MSCs, AMSCs, and DP-MSCs derived from five donors were studied by growth curve determination, the abilities of osteogenesis, chondrogenesis and adipogenesis, immunomodulatory function test, and Western blotting. Their results showed that MSCs derived from different types of placental tissue had different biological characteristics, and the same type of MSCs from different individuals were also heterogeneous. This suggests that preselection of placenta-derived MSCs with specific biological advantages may improve the efficacy of cell therapy. Similarly, due to the heterogeneity of MSCs, optimal MSCs should be selected to improve the therapeutic effect. In addition, different cell isolation techniques also can affect the purity and subsets of MSCs. Moreover, cell culture condition can have different effects on the expansion and status of MSCs, thus affecting their heterogeneity. With increasing age of donors, the abilities of multi-lineage differentiation, homing, immune regulation, and oxidative stress regulation of MSCs gradually decrease to disappear. This also verifies that the differences between senescent and young MSCs are closely related to senescence-related heterogeneity. Currently, MSCs have been demonstrated to have a wide range of therapeutic potential in several experimental models. However, MSCs are heterogeneous cell population, and this heterogeneity of MSCs makes them hindered in therapy [251]; its heterogeneity is also a limiting factor for the clinical translation of MSCs therapy [253]. Therefore, in order to avoid the differences in clinical treatment caused by the heterogeneity of MSCs, firstly, MSCs should be obtained by using standardized cell preparation methods to avoid heterogeneity associated with culture conditions and cell isolation procedures. Then, the heterogeneity of MSCs can be analyzed by advanced single-cell RNA sequencing (scRNA-seq), and the implementation of a comprehensive single-cell map will help to better solve the heterogeneity [251]. In addition, appropriate pretreatment of the cell culture medium or genetic manipulation may change the characteristics and therapeutic potential of MSCs, which will play a key role in the future clinical MSCs therapy, through the different treatments of MSCs, personalize medicine for patients. In the future, we need to find a better method to accurately analyze the heterogeneity of MSCs, so as to better solve the differences in therapeutic effects caused by heterogeneity and help to select the most effective MSCs for clinical research and treatment of diseases to obtain the maximum therapeutic effect.

According to previous studies, there are ethical and safety issues in the clinical transformation with stem cells [256]. Firstly, MSCs have a risk of generating tumors because of their ability of self-renewal. A study indicated that analyzing the 42 studies and 32 reports, it was finally concluded that MSCs promoted cancer metastasis and occurrence [257]. Then, MSCs have a risk of the formation of thrombus. In a phase I/IIa study of the use of WJ-MSCs to treat osteoporotic vertebral fractures, the complications that may be related to MSCs developed, such as pulmonary embolism and tumor formation, which suggested that we should pay attention to the complications that may occur during the clinical application of MSCs [156]. An article reported that MSCs can promote the development and progression of cancer in various ways; however, MSCs can also participate in the induction and inhibition of cancer progression and metastasis mediated by some signaling pathways such as PI3K/AKT signaling pathway, JAK/STAT signaling pathway, Wnt signaling pathway, Hippo signaling pathway, MYC signaling pathway, and NF-κB signaling pathway [258]. Therefore, for the safety of MSCs therapy, long-term follow-up is needed to assess the oncologic safety of MSCs for widespread clinical application in the future [259]; furthermore, how to circumvent the pro-tumor effects of MSCs needs to be studied, which will provide a safe basis for the large-scale clinical translation of MSCs.

## Conclusion and outlook

Senescence is age-related, which is characterized by cellular senescence and accompanied by increased inflammation. Senescence can cause different age-related diseases, such as osteoporosis, neurodegenerative diseases, POF, CVDs. Thus, it can be seen that senescence is very harmful to human body. At present, it has been proved that exercise, appropriate nutritional intervention, CR, DR, maintenance of iron homeostasis, and some drug therapies can alleviate senescence. However, these therapeutic effects are not as good as we expected; deeper understanding the cause of senescence and exploring more effective measures to delay or even reverse senescence are of great significance. Notably, aside from telomere shortening, DNA damage, mitochondrial dysfunction, epigenetic changes, and oxidative stress may contribute to senescence, endogenous stem cell exhaustion may also be involved in the process of senescence.

Stem cell-based cell therapy is becoming increasingly popular, especially MSCs. MSCs can be obtained from a variety of tissues and have strong abilities of self-renewal and differentiation. In addition, MSCs can secrete cytokines and exert immune properties, which make them have a great prospect in the treatment of senescence. MSCs can improve the functions of various organs to achieve anti-senescence effect, which may be related to the fact that MSCs secrete soluble factors to affect the survival and proliferation of surrounding cells. UC-MSCs represent an attractive and ethical cell source for stem cell therapy [260]. On the one hand, they are easy to extract materials, wide sources, isolated and cultured in vitro, stable biological properties, low tumorigenicity and immunogenicity. On the other hand, culturing MSCs from umbilical cord does not have ethical problems, does not cause additional pain to the patient, and does not easily lead to the spread of the disease in the population [247]. It seems to be a promising kind of MSCs for the treatment of senescence. At present, there have been some clinical trials based on MSCs in the treatment of senescence and senescence-related diseases, which have preliminarily proved the efficacy and safety of MSCs therapy.

Overall, it is of interest that MSCs therapy has great therapeutic prospects in regenerative medicine; however, it is far way to achieve satisfactory anti-senescence efficacy of MSC-based therapy. Therefore, it is great of potentials to reinforce therapeutic efficacy by specific gene modification in MSCs. The engineered gene-modified MSCs have more advantages, such as long-term and efficient expression of targeted molecules or effector molecules to enhance higher therapeutic effects. In addition, due to their targeting properties, MSCs are also very suitable to be used as vectors to deliver genes to the site of injury [261]. IL-10 is an anti-inflammatory cytokine, mainly secreted by immune cells, which can limit the proliferation of T cells and inhibit the production and expression of pro-inflammatory factors such as IL-2, IFN-γ, IL-6, TNF-α [262]. Overexpressing IL-10 MSCs were found to increase autophagy and protect rats from neuronal damage induced by traumatic brain injury [263]. There were many similar studies regarding IL-10-modified MSCs showing better therapeutic effects than naive MSCs. For example, IL-10 -modified human amniotic MSCs showed better effects in accelerating wound healing, promoting angiogenesis, regulating inflammation, and promoting extracellular matrix remodeling to promote wound healing and improve the quality of healing [261]. Human IL-10-modified UC-MSCs also showed successfully alleviated high-fat diet (HFD) that induced the obesity in mice [60]. In the future, this kind of genetic engineering MSCs may provide a new idea and inspiration for clinical treatment of senescence.

In summary, MSC-based therapy is a promising cell therapy for anti-senescence and will make a great contribution to anti-senescence in the future. Specific gene-modified MSCs may have much better effects on anti-senescence and related diseases.

## Data Availability

Not applicable.

## Abbreviations

MSCs: Mesenchymal stem cells
AD: Alzheimer’s disease
PD: Parkinson’s disease
MS: Multiple sclerosis
CVDs: Cardiovascular diseases
CR: Caloric restriction
DR: Dietary restriction
ROS: Reactive oxygen species
SASP: Senescence-associated secretory phenotypes
GCA: Granular calcains
RER: Respiratory exchange ratio
CNS: Central nervous system
GFAP: Glial fibrillary acidic protein
ARC: Arcuate nucleus
VMH: Ventromedial hypothalamic nucleus
LH: Lateral hypothalamic nucleus
h&E: Hematoxylin and eosin
MT: Masson's trichrome
TSC: Totipotent stem cell
HFSCs: Hair follicle stem cells
NFATc1: Nuclear factor of activated T cell cytoplasmic 1
BMSCs: Bone marrow mesenchymal stem cells
hUC-MSCs: Human umbilical cord mesenchymal stem cells
ALS: Amyotrophic lateral sclerosis
ASCs: Adipose tissue-derived stem cells
ADSCs: Adipose-derived mesenchymal stem cells
SAMP: Senescence-accelerated mouse/prone
TBI: Total body irradiation
MPCs: Mesenchymal progenitor cells
AMSCs: Amniotic membrane mesenchymal stem cells
HGPS: Hutchinson–Gilford progeria syndrome
allo-hMSCs: Allogeneic human mesenchymal stem cells
BMD: Bone mineral density
OVCFs: Osteoporotic vertebral compression fractures
WJ-MSCs: Wharton's jelly-derived mesenchymal stem cells
MI: Myocardial infarction
mGSCs: Multipotent germline stem cells
ES: Embryonic stem cells
RLP: Residual particle
VEGF: Vascular endothelial growth factor
AT-MSCs: Adipose tissue mesenchymal stromal cells
AMI: Acute myocardial infarction
LVEF: Left ventricular ejection fraction
LVESV: Left ventricular end-systolic volume
LVEDV: Left ventricular end-diastolic volume
NSCs: Neural stem cells
NAD^+^: Nicotinamide adenine dinucleotide
MuSCs: Muscle stem cells
McSCs: Melanocyte stem cells
NR: Nicotinamide riboside
POF: Premature ovarian failure
HA: Hyaluronic acid
HESC-MSCs: Human embryonic stem cells
WS: Werner syndrome
CKD: Chronic kidney disease
CWJ-MSCs: Camel Wharton jelly mesenchymal stem cells
COPD: Chronic obstructive pulmonary disease
CRP: C-reactive protein
AMSCs: Amniotic membrane mesenchymal stem cells
IL-10: Interleukin-10
MSC-CM: Mesenchymal stromal cell-conditioned medium
HG: High glucose
EVs: Extracellular vesicle
MMP-1: Matrix metalloproteinase-1
MMP-3: Matrix metalloproteinase-3
Y-CM: CM derived from young MSCs
CP-MSCs: Chorionic plate mesenchymal
DP-MSCs: Decidual plate mesenchymal stem cells
scRNA-seq: Single-cell RNA sequencing
HFD: High-fat diet
